# Neuraxial versus general anesthesia in elderly patients undergoing hip fracture surgery and the incidence of postoperative delirium: a systematic review and stratified meta-analysis

**DOI:** 10.1186/s12871-023-02196-9

**Published:** 2023-07-22

**Authors:** Karis Yui-Lam Cheung, Timothy Xianyi Yang, David Yew-Chuan Chong, Eric Hang-Kwong So

**Affiliations:** Queen Elizabeth Hospital, Tertiary Hospital in Kowloon, 30 Gascoigne Road, Hong Kong, Hong Kong

**Keywords:** Neuraxial anesthesia, General anesthesia, Hip fracture, Postoperative delirium

## Abstract

**Background:**

Evidence-based effect of anesthetic regimens on postoperative delirium (POD) incidence after hip fracture surgery is still debated. Randomized trials have reported inconsistent contradictory results largely attributed to small sample size, use of outdated drugs and techniques, and inconsistent definitions of adverse outcomes. The primary objective of this meta-analysis was to investigate the impact of different anesthesia regimens on POD, cognitive impairment, and associated complications including mortality, duration of hospital stay, and rehabilitation capacity.

**Methods:**

We identified randomized controlled trials (RCTs) published from 2000 to December 2021, in English and non-English language, comparing the effect of neuraxial anesthesia (NA) versus general anesthesia (GA) in elderly patients undergoing hip fracture surgery, from PubMed, EMBASE, Google Scholar, Web of Science and the Cochrane Library database. They were included if POD incidence, cognitive impairment, mortality, duration of hospital stay, or rehabilitation capacity were reported as at least one of the outcomes. Study protocols, case reports, audits, editorials, commentaries, conference reports, and abstracts were excluded. Two investigators (KYC and TXY) independently screened studies for inclusion and performed data extraction. The risk of bias was assessed using the Cochrane Collaboration risk-of-bias tool. The quality of the evidence for each outcome according to the GRADE working group criteria. The odds ratio (OR) and 95% confidence intervals (CI) were calculated to assess the pooled data.

**Results:**

A total of 10 RCTs with 3968 patients were included in the present analysis. No significant differences were found in the incidence of POD comparing NA vs GA [OR 1.10, 95% CI (0.89 to 1.37)], with or without including patients with a pre-existing condition of dementia or delirium, POD incidence from postoperative day 2–7 [OR 0.31, 95% CI (0.06 to -1.63)], in mini-mental state examination (MMSE) score [OR 0.07, 95% CI (-0.22 to 0.36)], or other neuropsychological test results. NA appeared to have a shorter duration of hospital stay, especially in patients without pre-existing dementia or delirium, however the observed effect did not reach statistical significance [OR -0.23, 95% CI (-0.46 to 0.01)]. There was no difference in other outcomes, including postoperative pain control, discharge to same preadmission residence [OR 1.05, 95% CI (0.85 to 1.31)], in-hospital mortality [OR 1.98, 95% CI (0.20 to 19.25)], 30-day [OR 1.03, 95% CI (0.47 to 2.25)] or 90-day mortality [OR 1.08, 95% CI (0.53–2.24)].

**Conclusions:**

No significant differences were detected in incidence of POD, nor in other delirium-related outcomes between NA and GA groups and in subgroup analyses. NA appeared to be associated with a shorter hospital stay, especially in patients without pre-existing dementia, but the observed effect did not reach statistical significance. Further larger prospective randomized trials investigating POD incidence and its duration and addressing long-term clinical outcomes are indicated to rule out important differences between different methods of anesthesia for hip surgery.

**Trial registration:**

10.17605/OSF.IO/3DJ6C.

**Supplementary Information:**

The online version contains supplementary material available at 10.1186/s12871-023-02196-9.

## Background

Hip fractures are among the most frequent causes of hospitalization and disability in the geriatric population. Since 2010, an estimated 2.7 million patients a year were diagnosed with hip fractures [1], and this number has been projected to increase to over 6 million worldwide by 2050 [2].

Hip fracture is an acute surgical condition and approximately 98% of patients warrant operation requiring anesthesia [3]. As the geriatric group is often associated with frailty and multiple comorbidities, patients undergoing hip fracture surgery are at a significantly higher risk of perioperative mortality and morbidity, including POD, respiratory complications, myocardial ischemia, and cerebrovascular accidents [4–6].

Emphasis on multi-disciplinary efforts have been made over the last decade to improve geriatric hip fracture outcomes; however, evidence-based medicine in orthogeriatric anesthesia has yet to identify an optimal anesthetic technique for their management. Observational studies suggested NA is associated with lower 30-day mortality risk, in-hospital stay, readmission rates [7, 8], delirium [9], and major medical complications [10–13]. Contrarily, randomized trials have reported inconsistent contradictory results for the same outcomes depending on different anesthesia techniques; this was largely attributed to a reduced number of trial participants, small sample size, use of outdated drugs and techniques that are irrelevant to current practice, and inconsistent definitions of adverse outcomes [3].

POD, in particular, represents one of the most common postoperative complications in patients with hip fractures [14]. POD incidence has been reported in 16.9–28% of patients and has been associated with increased 30-day mortality risk, prolonged hospital stay, difficulty in regaining daily function, and a higher risk of future cognitive dysfunction [14–16]. The effects of NA versus GA on the incidence of POD in older patients undergoing hip fracture surgery are largely uncertain. Recent systemic reviews and meta-analyses found no evidence to suggest that anesthesia technique influences POD but noted that evidence-based results is lacking [17].

Recently, several comparative studies have been published [9–13] and two large RCTs investigating this outcome, namely the REGAIN trial [18] and the RAGA trial [19], have been performed. In light of the newly published data, we conducted a systematic review and stratified meta-analysis of the recent comparative studies to investigate whether NA or GA affected the incidence of POD and its associated clinical outcomes in elderly patients undergoing hip fracture surgery, including POD from day 2–7, postoperative MMSE score and neuropsychological test results, postoperative pain control, length of hospital stay, incidence of discharge to same preadmission residence, in-hospital mortality, 30-day and 90-day mortality rates.

## Study design and methodology

This study protocol was pre-registered on PROSPERO (The International Prospective Register of Systematic Reviews) and on OSF.io. The meta-analysis and systematic review were conducted according to PRISMA guidelines (Preferred Reporting Items for Systematic Reviews and Meta-Analyses) [20].

### Eligibility criteria

We applied a strict inclusion and exclusion criteria. We included all randomized controlled studies published in the last 20years, from the year 2000 to December 2021 comparing NA versus GA in elderly patients undergoing hip fracture surgeries if they included delirium as an outcome. We only included traditionally published journal articles. We excluded studies that did not report the primary outcomes, as well as grey literature such as non-traditional articles including study protocols, case reports, audits, editorials, commentaries, conference reports and abstracts. No language restriction was placed on our search.

### Information source and search strategy

Two authors (KYC and TXY) independently searched PubMed, EMBASE, Google Scholar, Cochrane Library, and Web of Science for relevant results, using a combination of specific keywords and terms, including “spinal anesthesia”, “regional anesthesia”, “neuraxial anesthesia’, “general anesthesia”, “hip fracture”, “surgery”, “delirium”, and others. The search was updated to December 31, 2022, and the full search strategy and results can be found in the Appendix. We then scanned the reference lists and citations of the studies and any other relevant systematic reviews for further results.

### Data extraction

Both authors (KYC and TXY) performed initial data extraction from the included RCTs into a predesigned data spreadsheet separately. This data was then combined, and any disagreements were adjudicated by the third author (ES). Including the outcome measures, we extracted the following data: year of publication, country, sample size, age, sex, American Society of Anesthesiologists (ASA) physical status, type of surgery, anesthesia techniques, adjunct interventions, including use of nerve blocks and sedation, provided definitions of postoperative delirium, inclusion or exclusion of patients with pre-existing dementia or delirium, day of baseline and postoperative assessment of delirium. Due to challenges in the translation of the full study, the data from one RCT reported in Japanese [21] was obtained from Guay et al. [3] instead. The patient characteristics, study characteristics as well as the outcome data were tabulated using standardized forms. The appendices of the studies were checked for any missing data and the authors of the studies were contacted for missing data.

### Outcomes

The outcomes evaluated were the incidence of postoperative delirium, postoperative delirium incidence from day 2–7, postoperative MMSE (mini-mental state examination) score and other neuropsychological tests results, postoperative pain control, length of hospital stay, incidence of discharge to same preadmission residence, in-hospital mortality, 30-day and 90-day mortality rates.

### Risk of bias and methodological quality assessment

Using the Cochrane Collaboration risk-of-bias tool [22–25], both KYC and TXY independently assessed all the studies and evaluated them for bias, including selection bias, including random sequence generation and allocation concealment, performance bias such as blinding of participants and personnel, detection bias such as blinding of outcome assessment, attrition bias due to incomplete outcome data and reporting bias. We categorized the risk of bias as high risk, low risk, or unclear. Any difference in opinion was adjudicated by a third author DYC. We also evaluated the quality of evidence for each outcome according to the Grading of Recommendations, Assessment, Development and Evaluation (GRADE) tool [26, 27].

### Statistical analysis and discussion

The risk of bias table and meta-analysis were performed using the latest version of RevMan (5.4), available on the Cochrane website [22–25]. If the data reported median and inter-quartile ranges, the data was converted to estimated mean and standard deviation using the method described by Wan et al. [28]. We utilized a random effects model (DerSimonian and Laird method) [29, 30] for summary estimates. Pooled estimates for dichotomous outcomes were presented as Odds ratios (ORs) with 95% confidence intervals (CIs), and for continuous outcomes, mean differences with standard deviations were used. For transparency and to balance between the potential for bias and the loss of precision when studies at high or unclear risk of bias were excluded, we stratified the studies by the summary of risk of bias and presented the estimates of the intervention effects from the studies at low risk of bias and from all studies. Studies were assessed for heterogeneity using Cochran’s Q test and I^2^ tests. Substantial heterogeneity was defined as I^2^ > 50%, and in case of significant heterogeneity, we performed robustness tests to determine the source of heterogeneity, as well as further post hoc subgroup analyses to look for confounders as means of investigating heterogeneous results [31, 32]. The risk of publication bias was assessed by funnel plots and Egger’s regression test [33–35].

We performed a trial sequential analysis (TSA), a cumulative meta-analysis method to control both type I and type II errors and determine if the results of the meta-analysis are reliable, utilizing TSA software version 0.9.5.10 Beta [36]. For the primary outcome, the calculation was based on an anticipated 5% relative risk reduction in the intervention group using a two-sided alpha of 0.05 and a power of 80%. We estimated the heterogeneity level and control-group event rates from the meta-analyses.

## Ethics

None required.

## Outcomes of meta-analysis

### Study identification and selection

**Fig. 1:**
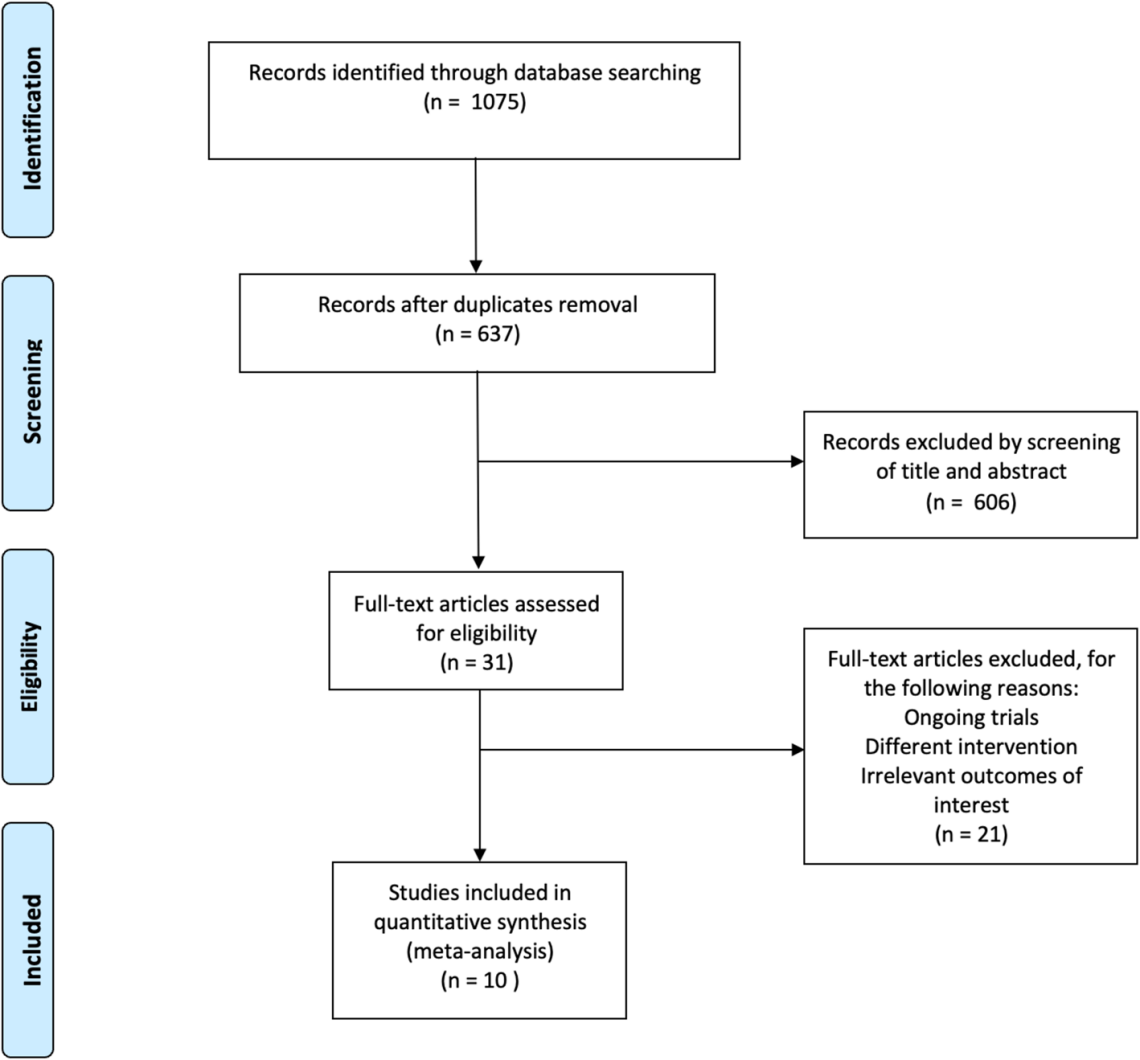
PRISMA study flow diagram. PRISMA = preferred reporting items for systematic reviews and meta-analysis

A total of 1075 studies were identified via database searches and screening of reference lists included in retained studies. 438 studies were removed for duplication, 606 papers were excluded by screening of titles and abstracts, 31 papers were assessed in detail, and 21 articles were excluded for the following reasons: ongoing trials, different intervention, and irrelevant outcomes of interest for this study. A total of 10 RCTs were finally included in this meta-analysis [18, 19, 21, 37–43]. The flow diagram of the study selection is shown in Fig. 1.

### Study characteristics

**Fig. 2:**
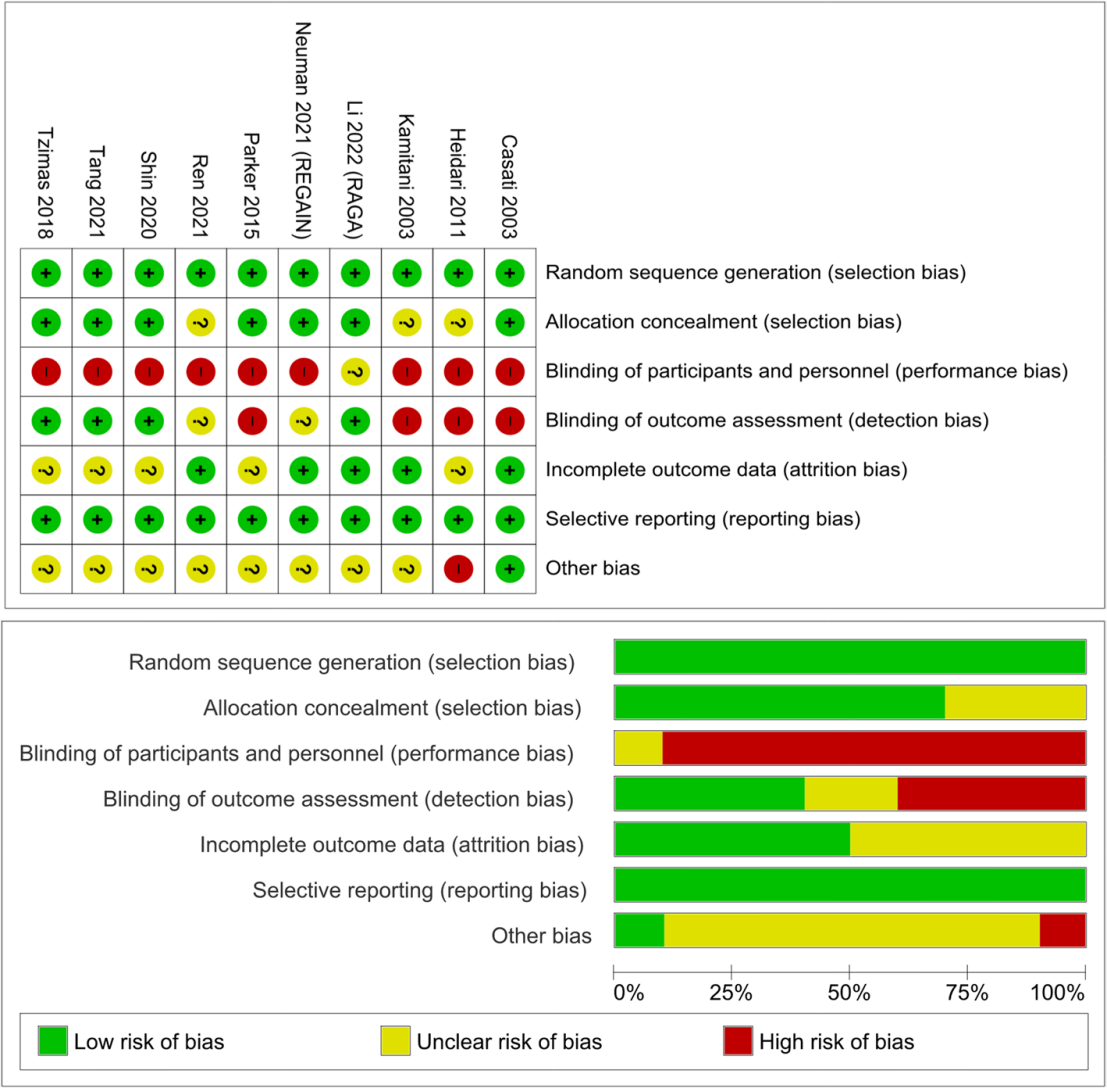
Risk of bias graph and summary for included studies. (“ + ” = low risk of bias; “-s” = high risk of bias; and “?” = unclear risk of bias)

The characteristics of each included RCT are summarized in Table 1 [18, 19, 21, 37–43]. The 10 studies included 3968 patients with hip fractures who received either NA (spinal, epidural, or combined spinal epidural) or GA. Five out of ten studies allowed for sedation if required or additional regional blocks. Patients over 40years old with an ASA status ranging from I to IV were included. All RCTs were conducted after the year 2000; six studies were conducted in Asia (China, Iran, Japan and Korea), three in Europe (UK, Greece, Italy), and one in North America (USA and Canada). Nine out of ten trials demonstrated low to moderate risk of bias except for blinding of participants and personnel (Fig. 2).

**Table 1:** Summary of characteristics of study

RCT trial	Country	Sample size (*N* = total) (male/female) [NA/GA]	Age range Avg age: (total) [NA/GA]	ASA status	Surgery type	Type of Anesthesia (NA vs GA)	Postoperative delirium definition	Pre-existing dementia / delirium patients	Postoperative delirium Baseline / postoperative day of assessment	Study outcome measures
**Li et al.** [19]** RAGA**	China	*N* = 942 (247 /695) [ 471/ 471]	71–82 (77) [77 / 77]	ASA I-IV I: 39 II: 713 III: 188 IV: 2	Closed reduction and internal fixation of femur Open reduction + internal fixation of femur	NA: SA / EA/ CSE; Type, dose, and use of nerve block were at discretion of anesthesiologist (No sedation) GA: IV induction agents, maintained with IV or inhalational agents *Adjunct regional blocks encouraged in both groups * Any medications known to impair cognitive function were prohibited	Defined by: Confusion Assessment Method (CAM)	Included	Baseline assessment conducted Postoperative D1-7	POD: Incidence, episodes, subtypes, severity of delirium; Postoperative cognitive dysfunction; Length of hospital stay; Pain control;30-day mortality; Intraoperative adverse events
**Neuman et al.** [18] **REGAIN**	United States	*N* = 1600 (528/ 1072) [ 795 / 805]	> 50 [77.7 / 78.4]	ASA I-IV I: 40 II: 499 III: 949 IV: 87	N/A	NA: Single shot SA (Sedation as needed; adjusted to ensure OAAS scale between 5 and 2) GA: Inhaled anaesthetic agent for maintenance *Choice of agent conforming to their usual practice, all other aspects of care determined by the clinical team	Defined by: Confusion Assessment Method (CAM)	Excluded	Baseline assessment conducted Postoperative D1-3	Mortality; Recovery of walking ability; Incidence of delirium; Length of hospital stay
**Tang et al.** [38]	China	*N* = 110 (55/55) [36/74]	> 65 [78/ 76.6]	ASA II – IV II: 42 III: 60 IV: 8	Osteosynthesis, Artificial femoral head replacement, Total hip replacement	NA: single shot SA; (4 ml of 0.25% hypobaric ropivacaine) (Sedation with propofol; maintained BIS 60–80) GA: combined lumbar sacral plexus block (total 40 ml 0.25% ropivacaine) + 5* μg* IV sufentanil for pain relief Induction: propofol (1–1.5 mg/kg), sufentanil (0.1–0.2 μg/kg), and cis- atracurium (0.2 mg/kg) Maintenance: propofol with effect site concentration adjusted to maintain depth of sedation (BIS: 60–80)	Defined by Confusion Assessment Method (CAM)	Excluded	Baseline assessment conducted Postoperative D1-7 or until discharged	Postoperative delirium; 30-day function status; Pain scores; In-hospital cost; Major postoperative complications
**Ren and Wu** [37]	China	*N* = 281 (130/151) [154/ 127]	65–79 (74.12 ± 4.15) [73.12 ± 6.15 / 74.12 ± 4.15]	ASA I-III I: 122 II: 126 III: 33	N/A	NA: Single shot SA (20 μg fentanyl/kg and 0.75% ropivacaine, exact dosage not specified) (sedation not specified) GA: Induction with fentanyl 3.5ug/kg and propofol 1.5 mg/kg, Maintenance agent not specified	Not well defined and no actual data provided for this outcome	Excluded	Baseline assessment – not specified Postoperative D1-7	Postoperative delirium; cognitive impairment; Activities of Daily Living
**Shin et al.** [39]	Korea	*N* = 176 (46/130) [ 58/ 118]	71–88 [81.6/ 80]	N/A No difference in comorbidities among patient groups	Hemiarthroplasty (bipolar) / Internal fixation of femur	NA: Single shot hyperbaric bupivacaine (Dose dependent on height, Height < 160 cm: 9 mg, Height ≥ 160 cm: 11 mg) (sedation as required; midazolam starting at dose 0.02 mg/kg) GA: *TIVA group* – Induction: propofol, remifentanil, cis-atracurium (exact dosage not specified) Maintenance: TCI propofol (Marsh) and TCI remifentanil (Minto), BIS 40–60 *Inhaled group* – Induction: pentothal sodium, cis-atracurium, remifentanil (exact dosage not specified) Maintenance: Desflurane in oxygen-air mixture (40:60) + remifentanil infusion	Defined by: aggravation or new onset delirium after surgery Assessment method N/A	Included	Baseline assessment conducted N/A	Post-inflammatory marker changes; Mortality; Morbidity
**Tzimas et al.** [40]	Greece	*N* = 70 (N/A) [37/33)	> 65 (76)	ASA I-III (ASA I: 2 ASA II: 41 ASA III: 27)	N/A	NA: single shot SA (fentanyl 20 mcg and ropivacaine 0.75%, exact dosage not specified); (No sedation) GA: Induction: fentanyl 3–5 mg/kg and propofol 1.5 mg/kg, rocuronium 0.6 mg/kg Maintenance: Desflurane	Defined by: Confusion Assessment Method (CAM)	Excluded	Baseline assessment conducted Postoperative D1-4	Postoperative cognitive dysfunction; Incidence of delirium
**Parker and Griffiths** [41]	United Kingdom	*N* = 332 (87/ 235) [158/ 163]	52–105 (83) [82.9 / 83.0]	ASA I-II	Arthroplasty, Sliding hip screw/ plate & screws, Intramedullary nail	Exact technique and dose of drugs was the choice of the anesthetist	Defined by: 10-question mental test score	Included	Baseline assessment conducted N/A	Mortality; General complications; Postoperative delirium; Length of stay; Surgical outcome
**Heidari et al.** [42]	Iran	*N* = 387 (257/130) [190/197]	> 30	ASA I-III	N/A	NA: SA/ EA SA: plain bupivacaine 0.5% (3 ml) EA: plain bupivacaine 0.5% (total 25 ml) with epinephrine (1:200,000) (No sedation) GA: halothane + nitrous oxide Induction: fentanyl (2 μg/kg), thiopental (5 mg/kg); lidocaine (1.5 mg/kg); pancuronium (0.1 mg/kg) Maintenance: halothane (0.5–1.5%) in oxygen and nitrous oxide (ratio 1:1)	Defined by: new postoperative cognitive dysfunction (disorientation to time, place or person)	Excluded	Baseline assessment conducted Postoperative D1-2	Postoperative cognitive dysfunction; 30-day mortality; General surgical Complications; Pain relief;
**Casati et al.** [43]	Italy	*N* = 30 (2/28) [15/15]	67–94 (84) [ 84 / 84]	ASA II-III II: 13 III: 17	Hemiarthroplasty	NA: single shot spinal anesthetic (hyperbaric bupivacaine 7.5 mg 0.5%) (No sedation) GA: Sevoflurane Volatile induction (inspired concentration 5%) Maintenance anesthesia: sevoflurane	Defined by: Decline in MMSE score from baseline ≥ 2 points	Included	Baseline assessment conducted Postoperative D1-7	Postoperative cognitive dysfunction; Intraoperative hemodynamic changes and blood loss; Pain relief; PACU discharge time; Duration of hospital stay
**Kamitani et al.** [21]	Japan	*N* = 40 (21/19) [4/36]	> 70 ( 82)[83 / 81.4]	N/A Comparable physical status between GA and SA groups	N/A	NA: single shot SA (0.5% bupivacaine, exact dosage not specified) (No sedation) GA: sevoflurane + nitrous oxide	Defined by: Confusion Assessment Method (CAM)	Excluded	Baseline assessment – not specified Postoperative D1-4	Postoperative delirium; Length of surgery; hemodynamic status; analgesic requirements

*NA* neuraxial anesthesia, *GA* general anesthesia, *SA* spinal anesthesia, *CSE* combined spinal-epidural, *POD* postoperative delirium, *CAM* confusion assessment method, *ASA status* the American Society of Anesthesiologists Classification System, *MMSE* mini-mental state examination, *OAAS* Observer’s Assessment of Alertness/Sedation Scale, *N/A* not available, *PACU* post anesthesia care unit

### Postoperative delirium incidence

**Fig. 3:**
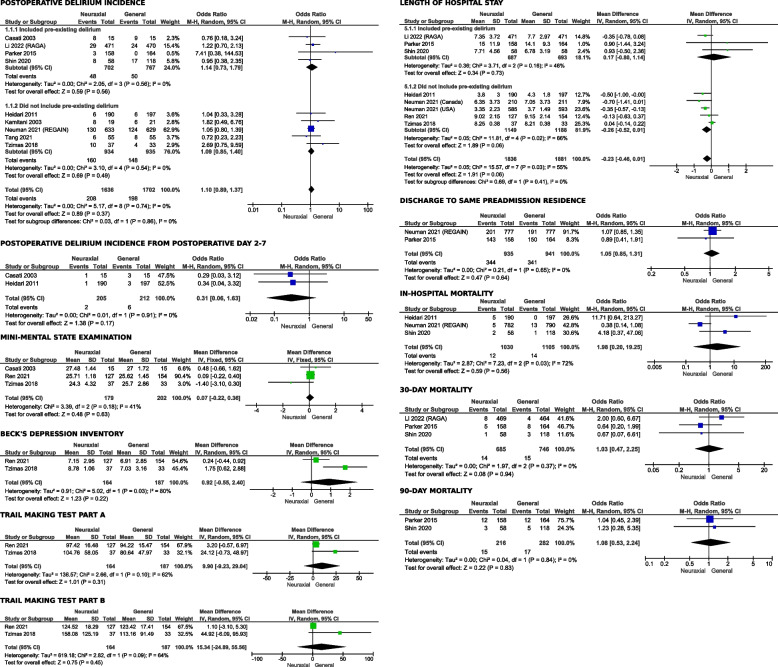
Forest plot for all study outcomes (MMSE = mini mental state of examination; NA = neuraxial anesthesia; GA = general anesthesia)

Nine studies [18, 19, 21, 38–43] which included 3227 patients reported POD incidence as an outcome. We found no significant difference in incidence of POD between the NA and GA groups as reported in Fig. 3 [OR 1.10, 95% CI (0.89 to 1.37)] (GRADE: High). There was also no overall significant heterogeneity with an I^2^ of 0% and Z-value was insignificant (*p* = 0.82). Subgroup analysis performed also showed no significant difference in POD incidence [I^2^ = 0%, Chi^2^ = 0.03, df = 1 (*p* = 0.86)] between the group of studies which included patients with pre-existing dementia and those who did not. Visual examination of the funnel plot (see appendix, eFig. 1) found no evidence of publication bias.

### Postoperative delirium incidence from day 2–7

Two studies [42, 43] including 417 patients reported incidence of POD persisting after postoperative day one to up to one week. No significant difference was found between NA and GA as reported in Fig. 3 [OR 0.31, 95% CI (0.06 to 1.63)] (GRADE: High). Both heterogeneity with an I^2^ of 0% and a Z-value of *p* = 0.17 were insignificant. Visual examination of the funnel plot (see appendix, eFig. 1) found no evidence of publication bias.

### Postoperative MMSE score

Three studies [37, 40, 43] including 381 patients also reported postoperative MMSE score as an outcome. There was no significant difference in the MMSE score between NA and GA groups as reported in Fig. 3 [OR 0.07, 95% CI (-0.22 to 0.36)] (GRADE: Moderate). There was moderate heterogeneity with an I^2^ of 41% and an insignificant Z-value (*p* = 0.63). No sensitivity analysis was performed given the small number of patients reported for this outcome. Visual examination of the funnel plot (see appendix, eFig. 1) found no evidence of publication bias.

### Postoperative neuropsychological tests

Two studies [37, 40] reported on postoperative neuropsychological tests including the Beck Depression Inventory, Trail-making Test Part A and B as outcomes, and a total of 351 patients were included. There were no significant differences between NA and GA groups for all three neuropsychological tests as shown in Fig. 3. For Beck Depression Inventory, the OR was 0.92, 95% CI (-0.55 to 2.40) (GRADE: Low). Visual examination of the funnel plot (see appendix, eFig. 1) found no evidence of publication bias. There was significant heterogeneity with I^2^ = 80%. For Trail-Making Test Part A, the OR was 9.90, 95% CI (-9.23 to 29.04) (GRADE: Low) and showed significant heterogeneity with I^2^ = 62%. For Trail-Making Test Part B, the OR was 15.34, 95% CI (-24.89 to 55.56) (GRADE: Low), and again there was significant heterogeneity with I^2^ = 64%. Z-values for all three tests were insignificant (*p* = 0.22 to 0.45). No sensitivity analysis was performed in consideration of the limited number of studies.

### Length of hospital stay

Seven studies [18, 19, 37, 39–42] including 3717 patients reported length of hospital stay. Although NA appeared to be associated with a shorter duration of hospital stay, especially in patients without pre-existing dementia or delirium, the observed effect did not reach statistical significance as reported in Fig. 3 [OR -0.23, 95% CI (-0.46 to 0.01)] (GRADE: Moderate). Heterogeneity was moderate with an I^2^ = 55% while Z-value was insignificant (*p* = 0.06). Subgroup analyses were performed and showed no significant difference. Visual examination of the funnel plot (see appendix, eFig. 1) found no evidence of publication bias.

### Incidence of postoperative discharge to same preadmission residence

Two studies [18, 41] that included 1876 patients reported incidence of postoperative discharge to same preadmission residence as an outcome. We found no significant difference in incidence of discharge to same preadmission residence between the NA and GA groups, as reported in Fig. 3 [OR 1.05, 95% CI (0.85 to 1.31)] (GRADE: High). There was no heterogeneity with I^2^ of 0% and an insignificant Z-value (*p* = 0.64) for overall effect. Visual examination of the funnel plot (see appendix, eFig. 1) found no evidence of publication bias.

### In-hospital mortality

Three studies [18, 39, 42] including 2135 patients reported on in-hospital mortality as an outcome. There was no significant difference in mortality rate between the NA and GA groups, as shown in Fig. 3 [OR 1.98, 95% CI (0.20 to 19.25)] (GRADE: Low). There was significant heterogeneity with I^2^ = 72% and Z-value was insignificant (*p* = 0.56). Sensitivity analysis showed Neuman 2021 as the source of the heterogeneity. Visual examination of the funnel plot (see appendix, eFig. 1) found no evidence of publication bias.

### 30-day mortality

Three studies [19, 39, 41] reported 30-day mortality as an outcome and included 1431 patients. We did not find any significant difference between the NA and GA groups with regard to 30-day mortality, as reported in Fig. 3 [OR 1.03, 95% CI (0.47 to 2.25)] (GRADE: Moderate). There was no significant heterogeneity with I^2^ = 0, and Z-value for overall effect was insignificant (*p* = 0.94). Visual examination of the funnel plot (see appendix, eFig. 1) found no evidence of publication bias.

### 90-day mortality

Two studies [39, 41] including 498 patients reported 90-day mortality rate as an outcome. There was no significant difference in 90-day mortality rate between the NA and GA groups, as reported in Fig. 3 [OR 1.08, 95% CI (0.53–2.24)] (GRADE: Moderate). There was negligible heterogeneity with I^2^ = 0 and Z-value was insignificant (*p* = 0.83). Visual examination of the funnel plot (see appendix, eFig. 1) found no evidence of publication bias.

### Postoperative pain control

Postoperative pain control was reported in 6 studies included in this meta-analysis [18, 19, 38, 40, 42–44] (see appendix, Table S1). However, the measuring tools and time of measurement of this outcome varied significantly across studies.

Heidari et al. [42] reported better pain relief immediately after surgery in the Post-Anesthesia Care Unit (PACU). Casati et al. [43] also reported better pain control in the NA group in the immediate postoperative period (1h) and at PACU discharge, but there was no significant difference in pain relief between the two groups 3h after the end of surgery. The REGAIN trial [18, 44], however, reported greater worst pain over the first 24 postoperative hours after surgery in the SA group (mean difference 0.40 [95% CI, 0.12 to 0.68]).

For pain control after the first 24 postoperative hours, Heidari et al. [42] reported no difference on the second and fifth postoperative days, while Tang et al. [38] and Tzimas et al. [40] also reported no significant difference in visual analogue scale (VAS) pain scores over 24h (*p* = 0.186) and the first 3days (*p* = 0.058 to 0.208) respectively. Li et al. [19] reported no significant difference in terms of worst pain score evaluated by VAS over the first 7 postoperative days. Contrarily, the REGAIN trial [18, 44] reported higher prescription analgesic use in the SA group at 60days (relative risk 1.33 [CI, 1.06 to 1.65]) compared to the GA group. Pain did not differ across groups at other time points, and overall satisfaction was similar across groups.

### Trial sequential analysis

Trial sequential analysis [36] is a methodology, similar to interim analyses performed in single randomized trials, that provides adjusted wider thresholds for significance in RCT when the required number of participants and the corresponding number of trials in a meta-analysis has not been reached. It can be used to decide whether a trial should be terminated early because of a sufficiently small P-value. It is utilized in meta-analyses to control random errors and to assess the need for further trials by weighing type I and II errors and to estimate when the effect is large enough to be unaffected by further studies, and it is a useful tool to assess the definitiveness of a meta-analysis. The cumulative z-statistic curve is drawn by adding the included studies chronologically. If this line does not cross the monitoring boundaries, there is likely no statistical significance and more studies are needed. If it remains in the inner wedge within the conventional boundaries, it is unlikely that further studies would change the effect. The required information size is a predefined calculation of the number of participants and events necessary to detect or reject an a priori assumed intervention effect.

TSA was performed for the findings of POD incidence, MMSE score, POD incidence from postoperative day 2–7, duration of hospital stay, incidence of discharge to same residence, and 30-day and 90-day mortality rates.

**Fig. 4:**
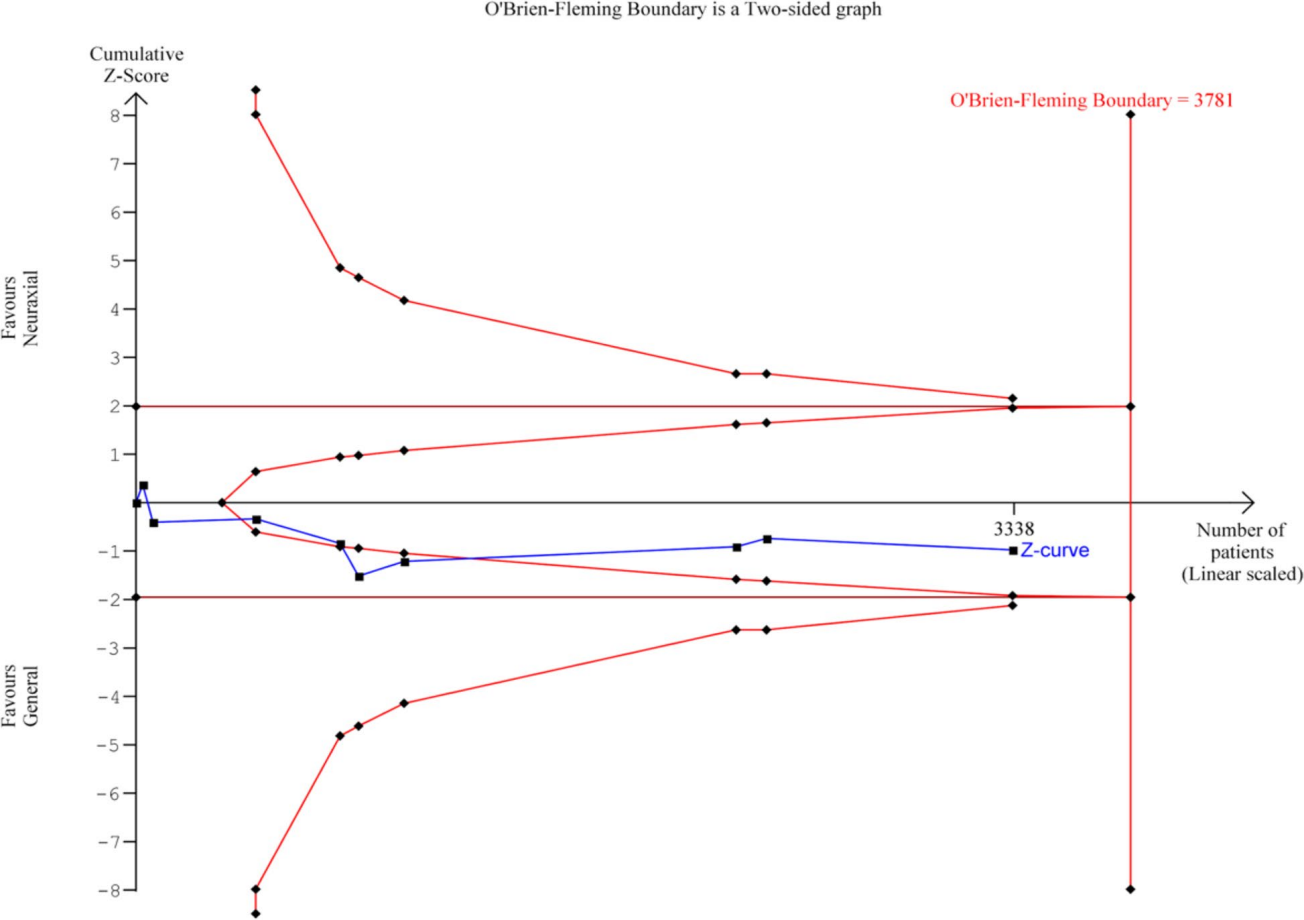
Trial sequential analysis of postoperative delirium incidence for neuraxial anesthesia versus general anesthesia

For incidence of POD in Fig. 4, the Z-curve (blue line) remained within the futility boundaries and did not reach the required information size. More randomized trials are required to reach definitive conclusions.

**Fig. 5:**
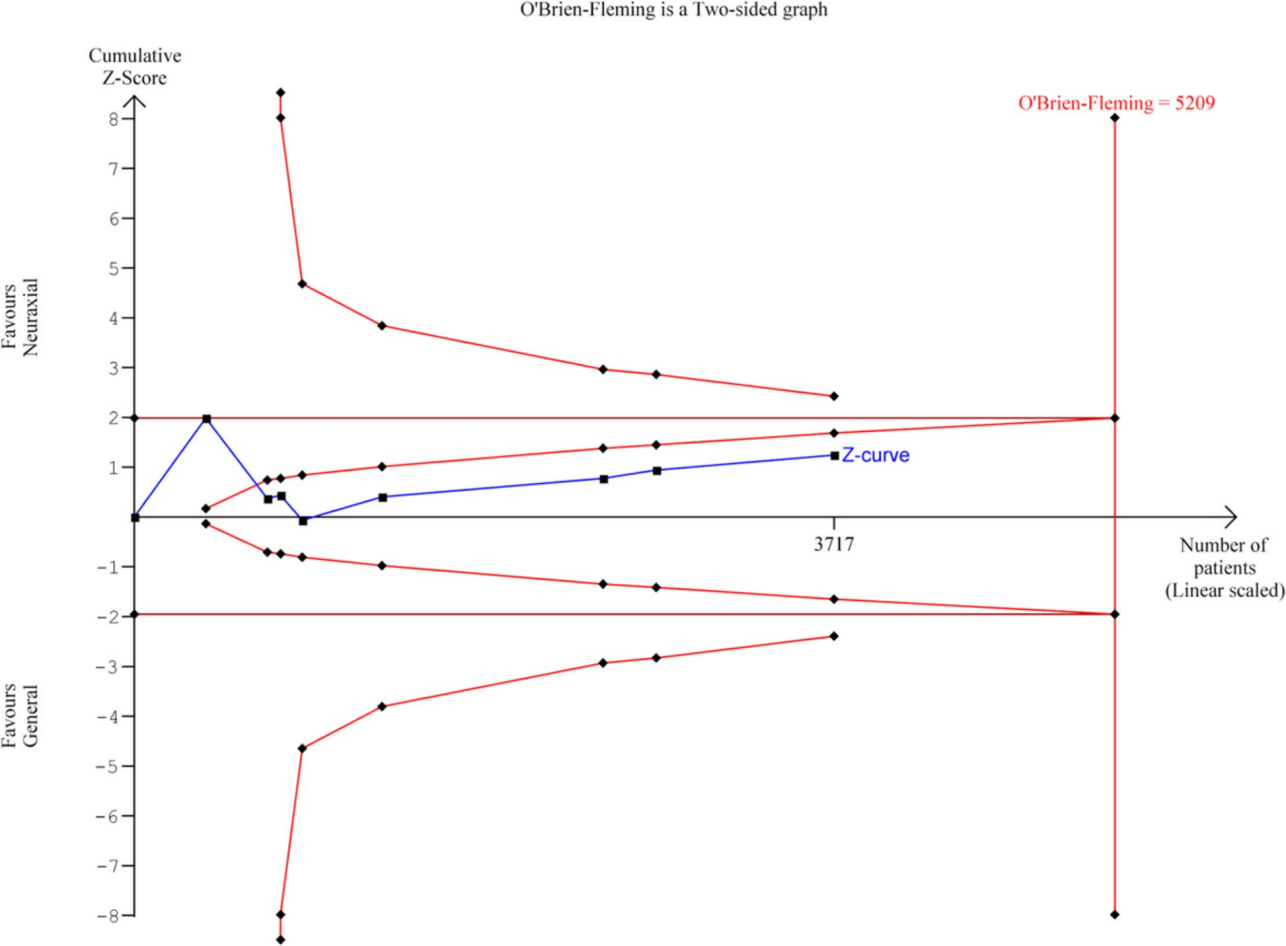
Trial sequential analysis of length of hospital stay for neuraxial anesthesia versus general anesthesia

The length of hospital stay favoured NA in the Forest plot although it was overall statistically insignificant. In the TSA analysis as shown in Fig. 5, it did not reach the required information size, hence no concrete conclusion can be drawn, and more trials are needed.

Regarding other outcomes including MMSE score, persistent POD from postoperative day 2–7, discharge to same preadmission residence and 30-day mortality rate, the Z-curve (blue line) remained within the boundaries for conventional benefit and monitoring boundaries, but remained outside the futility boundaries; thus we cannot conclude on the effect of NA and GA on these outcomes unless sufficient statistical power is achieved (see Appendix, eFigs. 2, 3, 4, 5, 6, and 7).

## Discussion

This meta-analysis was performed with a primary focus on investigating the impact of different anesthesia regimens on POD, cognitive impairment, and associated clinical implications including mortality, duration of hospital stay, and functional recovery.

In this meta-analysis, we showed there was no statistically significant difference in POD incidence between NA and GA groups. Subgroup analysis showed no difference in outcome either including or excluding patients with pre-existing dementia or preoperative delirium. TSA indicated the required information size was not reached and further large-sized randomized trials will be needed to reach definitive conclusions on this outcome. There were no significant differences in the other outcomes between the two anesthesia groups, but most were underpowered.

Our findings were similar to those of previous systematic reviews [17], and meta-analyses [3, 4]. However, Guay et al. [3] highlighted that most studies had poorly defined definitions of POD, and the time point of its evaluation was often unclear. These factors contributed to a widely varied outcome incidence and a statistically large heterogeneity among studies. Another concern was that most included studies were conducted before 2000 and referred to outdated clinical practices such as the use of long-acting benzodiazepines as induction agents [45]. Larger RCTs with standardized outcome definitions were recommended to eliminate the effect of such factors for recent analysis [3].

In response to the highlighted issues, this meta-analysis only included contemporary RCTs from 2000–2021 including 2 large multi-centre RCTs published in the past two years, the RAGA [19] and REGAIN trials [18], and included 3227 patients in the pooled analysis. Included RCTs provided a clear definition, despite not being uniform across considered studies, of POD and most used the CAM criteria [46] or other well-validated assessment tools for diagnosis of delirium. Nine out of ten trials demonstrated a low to moderate risk of bias except for blinding of participants and personnel. GRADE quality of evidence for seven out of eight reported outcomes were moderate to high. TSA was also performed to monitor for potential random errors and assess need for further trials.

Our study was able to demonstrate reduced heterogeneity for POD incidence compared to previous meta-analyses, which may have partially addressed the issue stemming from non-standardized outcome definitions and smaller sample size, resulting in a wide variability of reported outcomes. Although 5 RCTS used CAM test for its evaluation and 3 RCTS used other well-validated methods, 1 RCT [39] did not provide a clear definition of delirium nor information regarding its evaluation. Ren’s RCT [37] was not included in our evaluation of POD incidence because it did not have a clear definition nor actual data provided for this outcome. The information size was also not reached, indicating the need for further future trials with uniform and clear definitions of POD is essential. In addition, the development of and persistence of delirium is a multi-factorial process and confounding factors should be considered.

Pre-existing delirium or cognitive impairment is a predictor of postoperative delirium [14, 47]. Four RCTs, including the RAGA trial, adopted a broader inclusion criterion to include patients with preoperative dementia or delirium to enhance generalizability and reflect current practice. Five other studies excluded this group of patients to eliminate potential confounding factors. Subgroup analysis was conducted and showed no statistically significant difference in POD risk between these two subgroups.

The potential effect of sedatives given during NA and regional blocks performed during GA must also be considered [48–52]. More recent RCTS allowed the use of short-acting sedatives such as midazolam, if required. Both RAGA and REGAIN trials reported the use of regional blocks in both anesthetic groups. Although studies that prohibited sedatives did not find significant differences in POD incidence, this shift to multi-modal anesthesia regimens may mask any actual differences between NA and GA groups. Nevertheless, it is reflective of advancements in anesthesia practice and is more clinically relevant compared to previous meta-analyses.

Suboptimal pain control is another key factor to POD [14, 47, 53]. Although six studies reported on postoperative pain control, the measuring tools, time, and duration of pain score measurements varied significantly across studies, and data could not be pooled for analysis. Some studies reported better pain control in the NA group in the immediate postoperative period. The REGAIN trial, however, reported greater worse pain scores over the first 24 postoperative hours and higher prescription analgesic use at 60days for the NA group. A significant proportion of patients in both groups reported high levels of pain over the first 3 postoperative days (70% of enrolled patients). Contrary to other studies, the authors acknowledged this discrepancy and attributed it to possible undertreatment of pain for patients in the NA group, highlighting the need for additional efforts across both groups to better manage postoperative pain. Overall, none of the studies showed any significant difference in pain control between anesthetic regimens. Future studies reporting pain outcomes with standardized pain assessment methods, postoperative analgesic regimens, at standardized time points during the rehabilitation period would be beneficial for future analysis.

Regarding other delirium-related outcomes, there were no significant differences in POD prevalence after postoperative day one, MMSE scores, and postoperative cognitive functioning. Our study also did not find significant differences between groups for associated clinical implications including mortality and functional recovery during or after rehabilitation. However, most of these outcomes had limited data and were underpowered.

For duration of hospital stay, it approached statistical significance favouring NA in the Forest Plot, but the overall result was statistically insignificant. TSA showed that the required information size was not reached; hence more studies should be conducted.

We also took note of the recently published meta-analysis by Kunutsor et al. published in the BJA September 2022 issue [54] which compared several perioperative outcomes of NA vs GA for hip fractures including POD. Our findings were compatible with their data analysis results on common outcomes, including POD incidence, pain control, length of hospital stay (as proxy of being out of bed on the first postoperative day), incidence of return to preoperative preadmission residence, 30-day and 90-day mortality rates, but our meta-analysis focused more in detail on the effects of anesthesia regimens on POD incidence, neuropsychological functioning, and related clinical outcomes.

Considering the ongoing large Improve Hip Fracture Outcome in the Elderly Patient (iHOPE) trial [55] which intends to recruit 1032 patients, and thus reaching the required information size for POD based on our TSA, we hope more conclusive evidence could be made regarding POD and its associated outcomes to guide future clinical practice. Furthermore, we noted that the duration of POD was not well documented across studies, despite having significant impact on patient outcomes. Delirium-related implications including rehabilitation capacity or post-discharge to same preadmission residence were also not often evaluated. Inclusion of such outcomes in future trials are of great importance to guide future evidence-based practice.

Geriatric hip fracture anesthesia in the geriatric population remains a highly challenging part of anesthesia practice. Current studies and our meta-analysis have been unable to identify an ideal anesthetic regimen for hip fracture surgery, thus deliberate consideration should also be given to individual comorbidities, the expertise of anesthetists and surgeons, as well as patients’ wishes to achieve better care for our geriatric population.

## Limitations

One limitation of this study is the lack of statistical power for key outcomes. Despite including two recent large-scale RCTs, Forest plot analysis and TSA indicated that the required information size was not reached to make a conclusive judgement. Secondary delirium-associated outcomes were often reported only in a limited number of RCTs and were mostly underpowered.

While in this meta-analysis statistical heterogeneity for the primary outcome was insignificant, moderate heterogeneity was observed in a small number of secondary outcomes of our study. The Heidari trial [42] was a potential source for heterogeneity due to its inclusion of a younger hip fracture population (study inclusion criteria as age above 40years old). Nevertheless, this subgroup of patients below 50years old (total 85 participants) was only a small fraction of the total 3968 participants in the pooled analysis and hence would have likely had a minimal impact on the pooled analyses.

Furthermore, in this meta-analysis we noted that duration of POD was not well documented and its effect on postoperative functionality was also not well explored. Reporting on pain scores and postoperative pain regimens were also varied. Limited data were reported and a lack of standardized assessment tools prevented any pooled analysis and subgroup analyses or pooled analyses for the above-mentioned outcomes. The inclusion of such standardized outcomes would be desirable in future trials.

## Conclusions

This meta-analysis did not find any statistically significant difference in POD incidence between NA and GA groups or in any subgroup analyses. There was no difference in delirium incidence regardless of inclusion or exclusion of patients with pre-existing dementia or preoperative delirium. NA appeared to be associated with a shorter hospital stay, especially in patients without pre-existing dementia, but the observed effect did not reach statistical significance. There were no significant differences in other delirium-associated clinical outcomes. Larger prospective randomized trials with a uniform and clear definition of POD, outcomes on delirium prevalence or duration, rehabilitation and postoperative functionality are required.

## Supplementary Information

**Additional file 1.** 

## Data Availability

All data generated or analyzed during this study are included in this published article.

## Abbreviations

ASA: American Society of Anesthesiologists
CAM: Confusion assessment method
CSE: Combined-spinal epidural
CI: Confidence interval
EA: Epidural anesthesia
GRADE: The grading of recommendations, assessment, development, and evaluation methodology
GA: General anesthesia
MMSE: Mini mental state examination
NA: Neuraxial anesthesia
N/A: Not available
OAAS: Observer’s Assessment of Alertness/Sedation Scale
POD: Postoperative delirium
PONV: Postoperative nausea and vomiting
PRISMA: Preferred reporting items for systematic reviews and meta-analyses
RA: Regional Anesthesia
RCT: Randomized controlled trial
OR: Odds ratio
SMD: Standardized mean difference
TSA: Trial Sequential Analysis
