# Supplementary material for: Neuraxial versus general anesthesia in elderly patients undergoing hip fracture surgery and the incidence of postoperative delirium: a systematic review and stratified meta-analysis
Source: BMC Anesthesiol. 2023 Jul 22;23:250. doi: 10.1186/s12871-023-02196-9 (PMC10362612; doi:10.1186/s12871-023-02196-9)
Supplement: Supplementary file 1 — Additional file 1. [file 12871_2023_2196_MOESM1_ESM.docx]

**Appendix**

| **Supplementary material 1** | PRISMA checklist |
| --- | --- |
| **Supplementary material 2** | Full search strategy |
| **Supplementary material 3** | Summary of Postoperative Pain Outcome across included studies |
| **Supplementary material 4** | Funnel Plots for all outcomes |
| **Supplementary material 5** | TSA analysis for other outcomes |
| **Supplementary material 6** | GRADE analysis for outcomes |

**SUPPLEMENTARY MATERIAL 1**

PRISMA Checklist

| **Section and Topic** | **Item No** | **Checklist item** | **Location where item is reported** |
| --- | --- | --- | --- |
| **Title** | | | |
| Title | 1 | Identify the report as a systematic review. | Page 1 |
| **Abstract** | | | |
| Structured summary | 2 | Report an abstract addressing each item in the PRISMA 2020 for Abstracts checklist. | Page 1-2 |
| **Introduction** | | | |
| Rationale | 3 | Describe the rationale for the review in the context of existing knowledge. | Background |
| Objectives | 4 | Provide an explicit statement of the objective(s) or question(s) the review addresses. | Background |
| **Methods** | | | |
| Eligibility criteria | 5 | Specify the inclusion and exclusion criteria for the review and how studies were grouped for syntheses. | Study Design and Methodology |
| Information sources | 6 | Specify all databases, registers, websites, organisations, reference lists and other sources searched or consulted to identify studies. Specify the date when each source was last searched or consulted. | Study Design and Methodology |
| Search strategy | 7 | Present the full search strategies for all databases, registers and websites, including any filters and limits used. | Supplementary Material 2 |
| Selection process | 8 | Specify the methods used to decide whether a study met the inclusion criteria of the review, including how many reviewers screened each record and each report retrieved, whether they worked independently, and if applicable, details of automation tools used in the process. | Study Design and Methodology |
| Data collection process | 9 | Specify the methods used to collect data from reports, including how many reviewers collected data from each report, whether they worked independently, any processes for obtaining or confirming data from study investigators, and if applicable, details of automation tools used in the process. | Study Design and Methodology |
| Data items | 10a | List and define all outcomes for which data were sought. Specify whether all results that were compatible with each outcome domain in each study were sought (e.g. for all measures, time points, analyses), and if not, the methods used to decide which results to collect. | Study Design and Methodology |
|  | 10b | List and define all other variables for which data were sought (e.g. participant and intervention characteristics, funding sources). Describe any assumptions made about any missing or unclear information. | Study Design and Methodology |
| Study risk of bias assessment | 11 | Specify the methods used to assess risk of bias in the included studies, including details of the tool(s) used, how many reviewers assessed each study and whether they worked independently, and if applicable, details of automation tools used in the process. | Study Design and Methodology |
| Effect measures | 12 | Specify for each outcome the effect measure(s) (e.g. risk ratio, mean difference) used in the synthesis or presentation of results. | Study Design and Methodology |
| Synthesis methods | 13a | Describe the methods of handling data and combining results of studies, if done, including measures of consistency (such as I^2^ statistic) for each meta-analysis | Study Design and Methodology |
|  | 13b | Describe any methods required to prepare the data for presentation or synthesis, such as handling of missing summary statistics, or data conversions. | Study Design and Methodology |
|  | 13c | Describe any methods used to tabulate or visually display results of individual studies and syntheses. | Study Design and Methodology |
|  | 13d | Describe any methods used to synthesize results and provide a rationale for the choice(s). If meta-analysis was performed, describe the model(s), method(s) to identify the presence and extent of statistical heterogeneity, and software package(s) used. | Study Design and Methodology |
|  | 13e | Describe any methods used to explore possible causes of heterogeneity among study results (e.g. subgroup analysis, meta-regression). | Study Design and Methodology |
|  | 13f | Describe any sensitivity analyses conducted to assess robustness of the synthesized results. | Study Design and Methodology |
| Reporting bias assessment | 14 | Describe any methods used to assess risk of bias due to missing results in a synthesis (arising from reporting biases). | Study Design and Methodology |
| Certainty assessment | 15 | Describe any methods used to assess certainty (or confidence) in the body of evidence for an outcome | Study Design and Methodology |
| **Results** | | | |
| Study selection | 16a | Describe the results of the search and selection process, from the number of records identified in the search to the number of studies included in the review, ideally using a flow diagram. | Outcomes, Figure 1 |
|  | 16b | Cite studies that might appear to meet the inclusion criteria, but which were excluded, and explain why they were excluded. | Outcomes, Figure 1 |
| Study characteristics | 17 | Cite each included study and present its characteristics. | Outcomes, Table 1 |
| Risk of bias in studies | 18 | Present assessments of risk of bias for each included study. | Outcomes, Figure 2 |
| Results of individual studies | 19 | For all outcomes, present, for each study: (a) summary statistics for each group (where appropriate) and (b) an effect estimate and its precision (e.g. confidence/credible interval), ideally using structured tables or plots. | Outcomes, Figure 3, Table 1, Supplementary material 3 |
| Results of syntheses | 20a | For each synthesis, briefly summarise the characteristics and risk of bias among contributing studies. | Outcomes, Figure 2, Supplementary material 6 |
|  | 20b | Present results of all statistical syntheses conducted. If meta-analysis was done, present for each the summary estimate and its precision (e.g. confidence/credible interval) and measures of statistical heterogeneity. If comparing groups, describe the direction of the effect. | Outcomes, Figure 3 |
|  | 20c | Present results of all investigations of possible causes of heterogeneity among study results. | Outcomes, Figure 3 |
|  | 20d | Present results of all sensitivity analyses conducted to assess the robustness of the synthesized results. | Outcomes, Figure 3, |
| Reporting biases | 21 | Present assessments of risk of bias due to missing results (arising from reporting biases) for each synthesis assessed. | Outcomes, Figure 2 |
| Certainty of evidence | 22 | Present assessments of certainty (or confidence) in the body of evidence for each outcome assessed. | Outcomes, Figure 2, Supplementary Material 4, 6 |
| **Discussion** | | | |
| Discussion | 23a | Provide a general interpretation of the results in the context of other evidence. | Discussion |
|  | 23b | Discuss any limitations of the evidence included in the review. | Discussion |
|  | 23c | Discuss any limitations of the review processes used. | Limitations |
|  | 23d | Discuss implications of the results for practice, policy, and future research. | Discussion, Limitations |
| **Other information** | | | |
| Registration and protocol | 24a | Provide registration information for the review, including register name and registration number, or state that the review was not registered. | Other information |
|  | 24b | Indicate where the review protocol can be accessed, or state that a protocol was not prepared. | Other information |
|  | 24c | Describe and explain any amendments to information provided at registration or in the protocol. | Not applicable |
| Support | 25 | Describe sources of financial or non-financial support for the review, and the role of the funders or sponsors in the review. | Other information |
| Competing interests | 26 | Declare any competing interests of review authors. | Other information |
| Availability of data, code and other materials | 27 | Report which of the following are publicly available and where they can be found: template data collection forms; data extracted from included studies; data used for all analyses; analytic code; any other materials used in the review. | Other information |

**SUPPLEMENTARY MATERIAL 2**

Literature Full Search Strategy

| 1 exp hip fractures/  2 exp femoral fractures/ or femur fracture.mp.  3 femoral neck.mp. or exp femur neck/  4 regional an?esthesia.mp.  5 exp anesthesia, spinal/  6 neuraxial an?esthesia.mp.  7 general an?esthesia.mp  8 exp anesthesia, epidural/  9 exp anesthesia, general/  10 an?esthesia.mp.  11 exp delirium/  12 exp cognitive dysfunction/  13 exp cognitive impairment/  14 exp neurological function/  15 ("clinical trial" or "multicenter study" or "randomized controlled trial").pt. or (“comparative study” or “prospective” or “pilot” or “trial”).pt.  16 1 or 2 or 3  17 4 or 5 or 6 or 7 or 8 or 9 or 10  18 11 or 12 or 13 or 14  19 15 and 16 and 17  20 15 and 16 and 17 and 18  21 limit 17 to (adults, humans and year = "2003-Current")  * Searches were also made in the title and abstract fields  * Each part was translated for searching the other databases (Embase and Cochrane databases)  * The search was supplemented by hand-searching by checking the reference lists of journal articles  * Hand search was applied to Google Scholar to identify missing articles |
| --- |

**SUPPLEMENTARY MATERIAL 3**

Summary of postoperative pain outcome of included studies

**Table S1.** summary of pain outcomes

| RCT Trial | Postoperative analgesic regimen | Pain Assessment Method | Assessment Duration | Pain Scores |
| --- | --- | --- | --- | --- |
| Li et al. [19] RAGA | Postoperative analgesia was prescribed as per local practice.  Peripheral nerve block (single shot or continuous infusion) was highly recommended | Worst pain score by Visual Analogue Scale (VAS) over first postoperative 7 days from 0-100 | Postoperative 7 days | Presented as median score (IQR)  SA: 0 (0 to 20)  GA: 0 (0 to 10)  (no significant difference) |
| Neuman et al. [18]  REGAIN | All other aspects of care apart from SA/GA regimen were determined by the clinical team | Worst pain score over past 24 hours  Average pain score; and  Pain score at time of interview by Numerical Rating Scale (0 -10) | Postoperative Day 1 to Day 3 (assessed daily) or until discharge  Pain data (worst pain, average pain, at time of interview and prescription analgesics consumption) at 60, 180 and 365 days after randomization. | Worst pain in the past 24h before interview:  Presented as mean (SD)  Postoperative Day 1   - SA: 7.9 (2.6) - GA: 7.6 (2.8) - Adjusted mean difference 0.4 (0.12 to 0.68)   Pain did not differ across groups at other time points.  Satisfaction was similar across groups.  Prescription analgesic use at 60 days   - SA: 25% (141 of 563) - GA: 18.8% (108 of 574) - Relative risk 1.33 (CI, 1.06 to 1.65) |
| Tang et al. [37] | Patient controlled analgesia (PCA) – sufentanil (2.5ug/kg) and flurbiprofen axetil (10mg) in 100ml NS | VAS score:  Resting pain  Motion pain | Postoperative first 3 days | Presented as median score  Postoperative Day 1   - Resting pain: SA 0 / GA 1 (p value 0.131) - Motion pain SA 2/ GA 3 (p value 0.208)   Postoperative Day 2   - Resting pain: SA 0/ GA 1 (p value 0.132) - Motion pain: SA 2/ GA 3 (p value 0.058)   Postoperative Day 3   - Resting pain: SA 0 / GA 0 (p value 0.068) - Motion pain: SA 2 / GA 2 (p value 0.143) |
| Ren and Wu [36] | N/A | N/A | N/A | N/A |
| Shin et al. [38] | N/A | N/A | N/A | N/A |
| Tzimas et al. [39] | Anesthesia management was conducted according to routine clinical practice.  All patients were treated for postoperative pain to keep VAS score below 44 without any analgesic restriction. | VAS | Postoperative 24h | There were no significant differences between the two groups regarding the pain intensity using VAS score preoperatively or postoperatively. (p=0.186) |
| Parker and Griffiths [40] | N/A | N/A | N/A | N/A |
| Heidari et al. [41] | Intravenous morphine (5mg) to patients who requested analgesia | VAS (0-10)  Total amount of morphine consumption was recorded till 5 days after surgery | From discharge from recovery room, 2^nd^, 3^rd^ and 5^th^ day of surgery | Pain severity at recovery and 3^rd^ postoperative day was lower in NA group  Presented as mean +/- SD  Recovery room:   - SA 2.7 +/- 2.7; GA 4.8 +/- 3.1 - P value 0.00   2^nd^ day   - SA 3.8 +/- 2.3; GA 4.3 +/- 2.4 - P value 0.074   3^rd^ day   - SA 2.9 +/- 2.0; GA 3.4 +/- 1.9 - P value 0.020   5^th^ day   - SA 2.7 +/- 2.6 ; GA 2.9 +/- 2.1 - P value 0.137 |
| Casati et al. [42] | Preoperative analgesic: IV ketorolac 30mg every 8h  Postoperative analgesia (20 min before end of surgery):  Loading dose of IV tramadol 100mg  IV ketorolac 30mg  Ranitidine 50mg  Metoclopramide 10mg  Followed by continuous infusion of a combination of tramadol (12mg/h), ketorolac (3mg/h)  Pain treatment was continued for first 48h after surgery and then substituted with oral medications | Five-point verbal rating scale  1= no pain  2 = mild pain  3 = moderate pain  4 = severe pain  5 = unbearable pain | Recorded at PACU discharge  Recorded 1,3,6 and 12h after PACU discharge | Quality of pain control was better in SA group at PACU discharge and 1h after surgery  RR – p value = 0.14  1h – p value = 0.006  There was no difference in pain relief observed between the two groups 3h after surgery  3h – p value = 0.23  6h – p value = 0.18  12h – p value = 0.13 |
| Kamitani et al. [43] | N/A | N/A | N/A | N/A |

N/A = not available, VAS = visual analogue scale, SA = spinal anesthesia, GA = general anesthesia, PACU = post anesthetic care unit, SD = standard deviation

**SUPPLEMENTARY MATERIAL 4**

**eFig. 1.** Funnel plots for all outcomes

**SUPPLEMENTARY MATERIAL 5**

TSA analysis for other outcomes


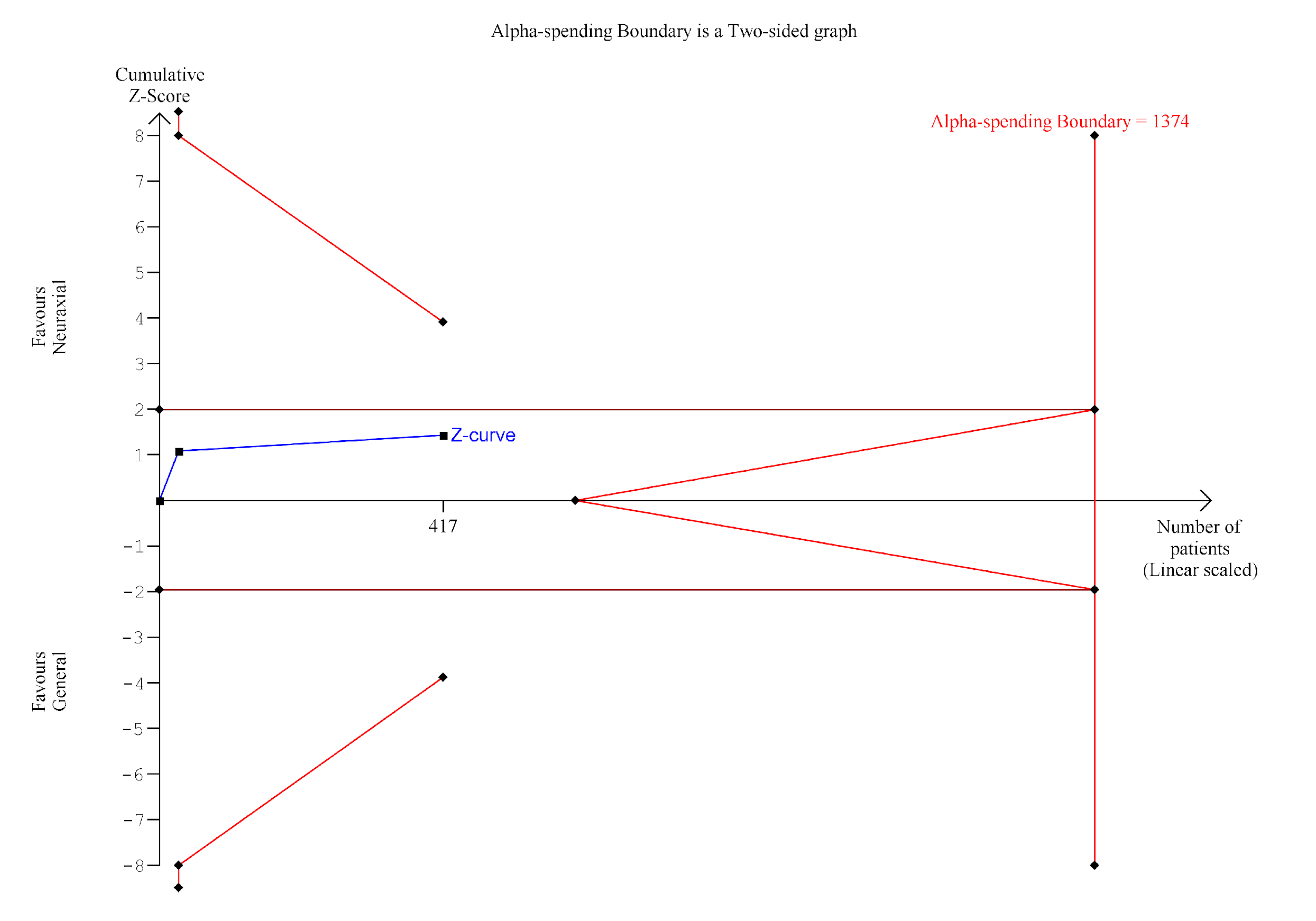


**eFig. 2.** Postoperative delirium incidence from postoperative day 2-7


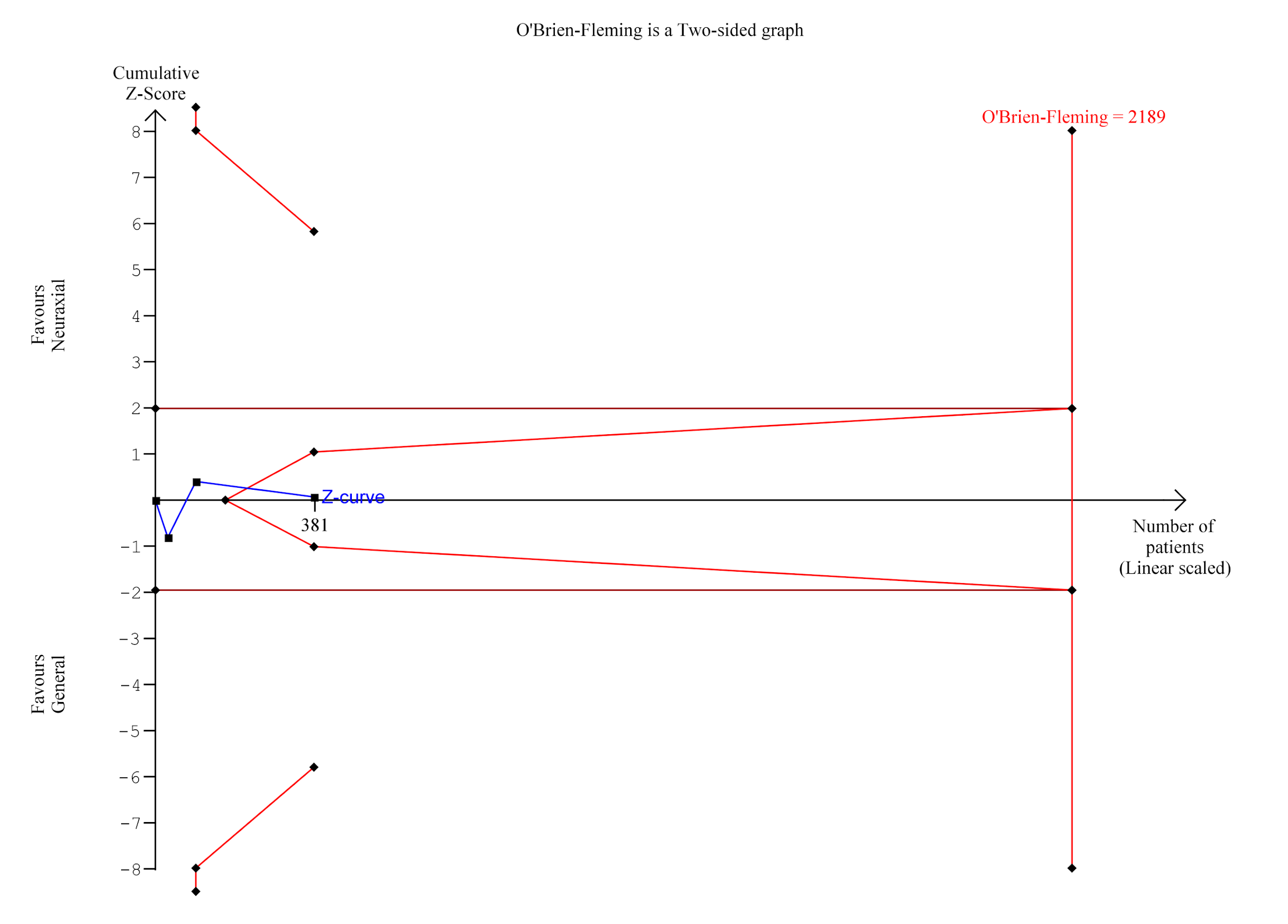


**eFig. 3.** Postoperative MMSE Score


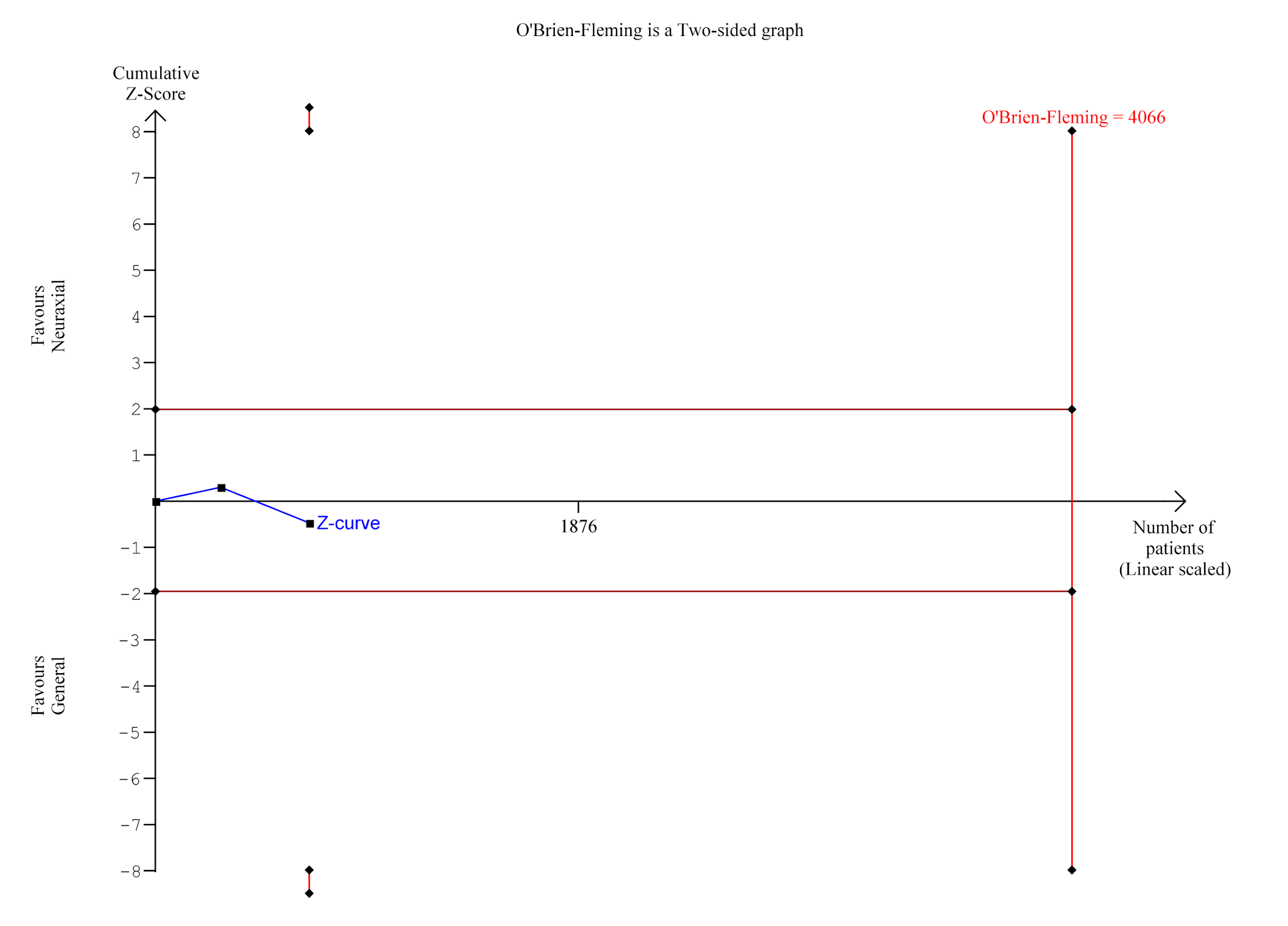


**eFig. 4.** Discharge to same preadmission residence


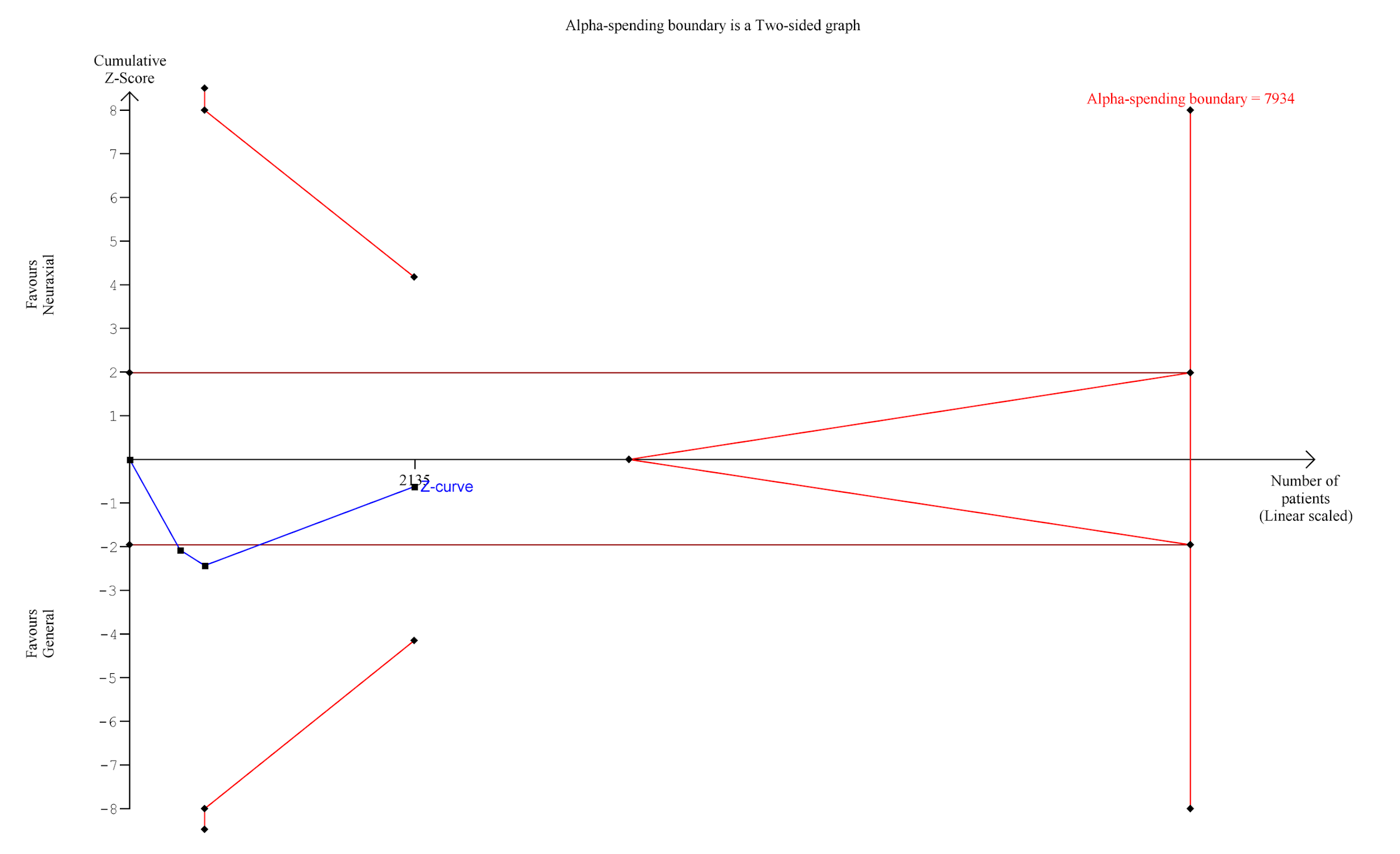


**eFig. 5.** In-hospital mortality


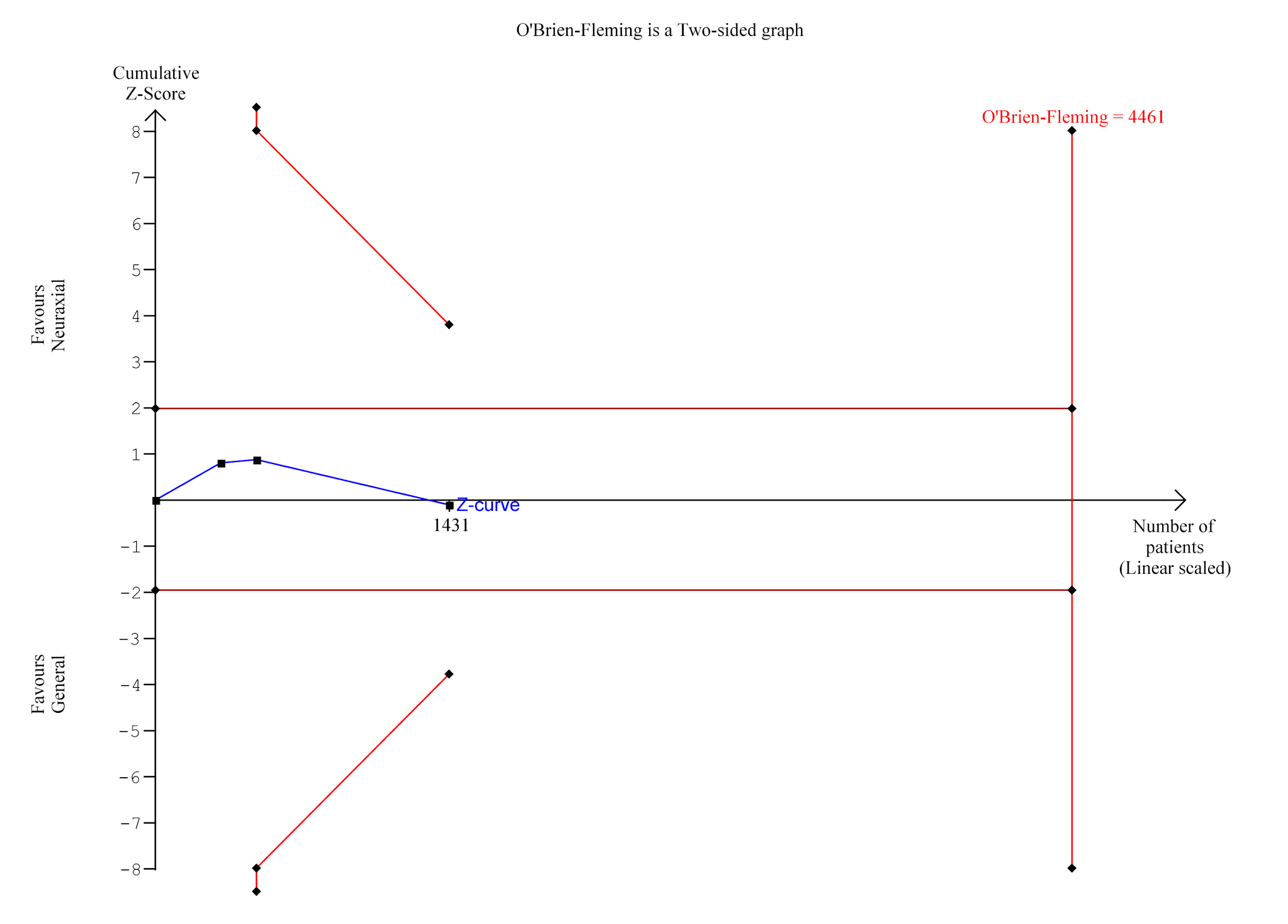


**eFig. 6.** 30-day mortality


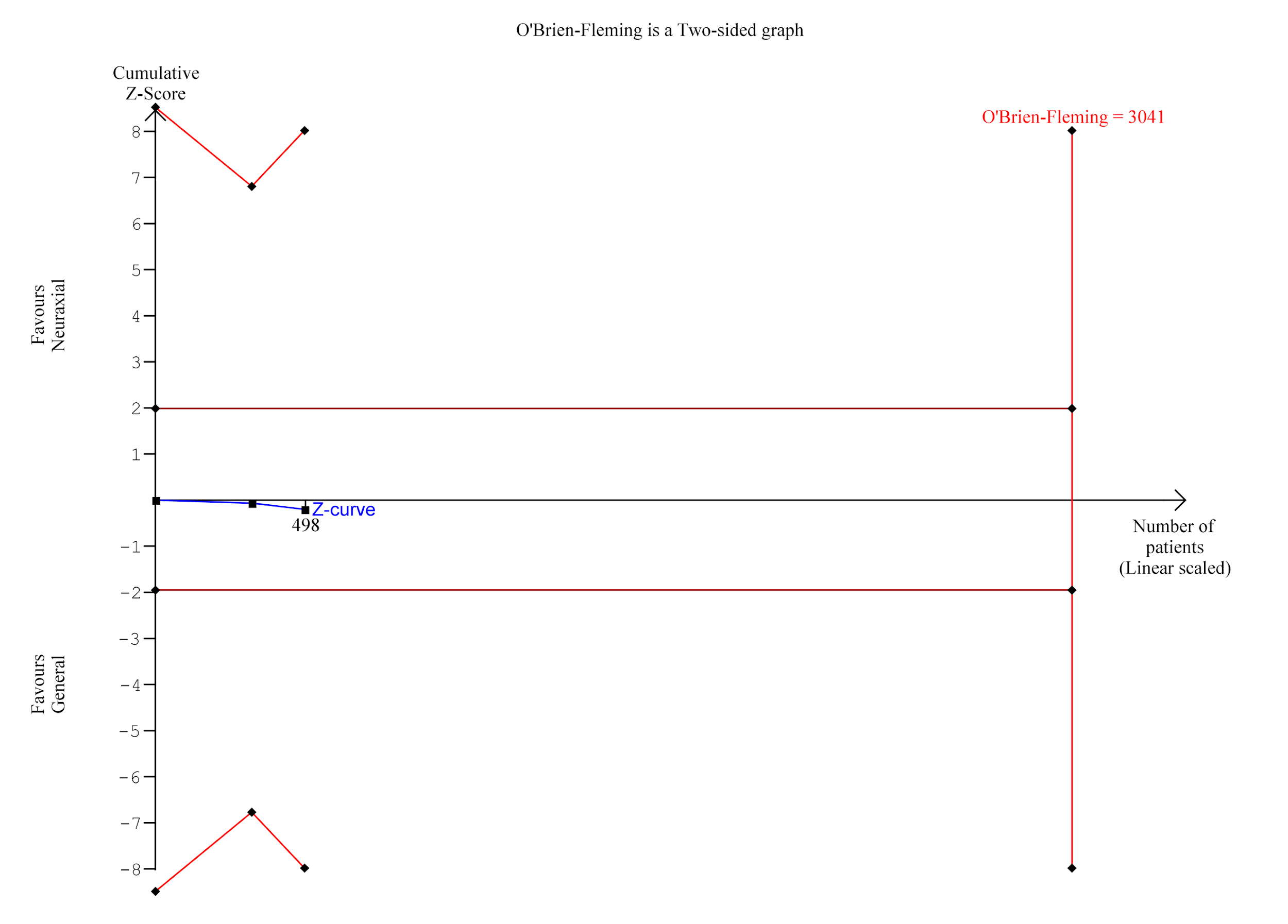


**eFig. 7.** 90-day mortality

**SUPPLEMENTARY MATERIAL 6**

GRADE analysis for outcomes

| Outcome | No. of participants (studies) | Risk of Bias | Inconsistency | Indirectness | Imprecision | Publication Bias | Overall Grading |
| --- | --- | --- | --- | --- | --- | --- | --- |
| Postoperative Delirium Incidence | 3338 (9 RCTs) | Low ^a^ | Insignificant ^b^ | Insignificant ^e^ | No serious imprecision | Undetected ^h^ | **High**  ⊕⊕⊕⊕ |
| Postoperative MMSE Score | 381 (3 RCTs) | Low ^a^ | Moderate ^c^ | Insignificant ^e^ | Likely ^g^ | Undetected ^h^ | **Moderate**  ⊕⊕⊕⊝  Due to imprecision and inconsistency ^c, g^ |
| Postoperative delirium incidence from postoperative Day 2-7 | 417 (2 RCTs) | Low ^a^ | Insignificant ^b^ | Insignificant ^e^ | No serious imprecision | Undetected ^h^ | **High**  ⊕⊕⊕⊕ |
| Postoperative Neuropsychological tests:   - Beck Inventory Depression - Trail Making Test Part A - Trail Making Test Part B | 351 (2 RCTs) | Low ^a^ | Significant ^d^ | Insignificant ^e^ | Likely ^g^ | Undetected ^h^ | **Low**  ⊕⊕⊝⊝  Due to imprecision and inconsistency ^d, g^ |
| Length of Hospital Stay | 3717 (7 RCTs) | Low ^a^ | Moderate ^c^ | Insignificant ^e^ | No serious imprecision | Undetected ^h^ | **Moderate**  ⊕⊕⊕⊝  Due to inconsistency ^c^ |
| Discharge to same preadmission residence | 1876 (2 RCTs) | Low ^a^ | Insignificant ^b^ | Insignificant ^e^ | No serious imprecision | Undetected ^h^ | **High**  ⊕⊕⊕⊕ |
| In-hospital mortality | 2135 (3 RCTs) | Low ^a^ | Significant ^d^ | Insignificant ^e^ | Likely ^f, g^ | Undetected ^h^ | **Low**  ⊕⊕⊝⊝  Due to imprecision and inconsistency ^d, f, g^ |
| 30-day mortality | 1431 (3 RCTs) | Low ^a^ | Insignificant ^b^ | Insignificant ^e^ | Likely ^f, g^ | Undetected ^h^ | **Moderate**  ⊕⊕⊕⊝  Due to imprecision ^f, g^ |
| 90-day mortality | 498 (2 RCTs) | Low ^a^ | Insignificant ^b^ | Insignificant ^e^ | Likely ^f, g^ | Undetected ^h^ | **Moderate**  ⊕⊕⊕  Due to imprecision ^f, g^ |

GRADE Working Group Quality of Evidence Grades

**High:** We are very confident that the true effect lies close to that of the estimate of the effect.

**Moderate:** We are moderately confident in the effect estimate: The true effect is likely to be close to the estimate of the effect, but there is a possibility that it is substantially different.

**Low:** Our confidence in the effect estimate is limited: The true effect may be substantially different from the estimate of the effect.

**Very Low:** We have little confidence in the effect estimate: The true effect may be substantially different from the estimate of effect

REASONS for grade assessment

1. All included studies were randomized controlled trials with standardized randomization. Nine out of ten trials demonstrated low to moderate risk of bias . Blinding of participants and personnel was not possible but binding of outcome assessors was adequate in 75% or more of the included studies.
2. I^2^ smaller than 30%
3. I^2^ ≥ 30- 60%
4. I^2^ > 60%
5. All outcomes included were based on direct comparisons, were performed on the population of interest, and were not surrogate outcomes.
6. Optimal information size or event size was not achieved
7. Wide 95% confidence interval
8. Either no evidence of a publication bias or correcting for the possibility of publication bias would not modify the conclusion.
